# Comparison of fetal middle cerebral arteries, umbilical and uterin artery color Doppler ultrasound with blood gas analysis in pregnancy complicated by IUGR

**Published:** 2013-01

**Authors:** Zahra Fardiazar, Simin Atashkhouei, Yousef Yosefzad, Mohamad Goldust, Reza Torab

**Affiliations:** 1 *Department of Obstetrics and Gynecology, Tabriz University of Medical Sciences, Tabriz, Iran.*; 2 *Department of Anesthesiology, Tabriz University of Medical Sciences, Tabriz, Iran.*; 3 *Student Research Committee, Tabriz University of Medical Sciences, Tabriz, Iran.*

**Keywords:** *Middle cerebral artery Doppler*, *Umbilical artery Doppler*, *IUGR*

## Abstract

**Background: **Fetal color Doppler is important for evaluation of hypoxia in intrauterine growth restriction (IUGR) fetus.

**Objective:** In this study we compare fetal and maternal color Doppler with blood gas analysis to detect fetal acidosis.

**Materials and Methods:** In this cross-sectional study we evaluated 100 hospitalized patients with IUGR for comparison of color Doppler results with arterial blood gas analysis. Results of Doppler sonography of fetus middle cerebral arteries, umbilical and uterine artery and umbilical artery ABG were studied in these neonates.

**Results:** Mean maternal age was 28±7 years, mean gestational age was 31.79±2.59 weeks and mean growth restriction was 3±2 weeks. Resistance increasing was observed in right uterine arteries of 37 mothers. It was normal in 60 mothers. Resistance increasing was observed in left uterine arteries of 36 mothers and nuch was seen in four cases. PCO_2_, PO_2_, and pH mean were 48.41±9.50 mmHg, 26.00±12.34 mmHg, and 7.28±0.10 in the neonates respectively.

**Conclusion:** In this study abnormal color Doppler in IUGR fetuses have no significant correlation with umbilical cord blood gas.

## Introduction

Intra-uterine growth restriction (IUGR) defined by body mass and weight loss to less than 10^th^ percentile is regarded as dangerous pregnancies due to complications resulted from delivery interventions in mother and later neonatal complications (1, 2). 

It is generally prevalent in 3-10% of the neonates. Causes of fetus growth retardation include: 1) Maternal factors such as poor nutrition which is the most common causes of IUGR but have the least risks. Mother’s hypertension is the most prevalent maternal factors associated with IUGR. 2) Factors related to placenta: In many cases of IUGR, the placenta is small and doesn't provide sufficient nutrition to the growing baby. In IUGR pregnancies, blood flow to the placenta decreases as pregnancy progresses, compared with normal pregnancy when blood flow to the placenta increases throughout pregnancy to meet the growing baby's demand for oxygen and nutrition. Cell death (apoptosis); in pregnancies complicated by IUGR, the placenta contains a relatively high proportion of cells that have a shorter life than normal. This means the placenta functions less well, thereby transferring fewer nutrients and less oxygen both to and from the baby and pre-eclampsia. 3) Factors related to fetus including major congenital anomalies (3, 4).

Prediction of IUGR especially existence of asphyxia in fetuses with growth restrictions is very important in starting preventive treatments, determining kind of delivery and treatment required by the neonate as well as parents’ information (5). 

Sonography is regarded a useful way in determining pregnancy age. It may have high sensitivity in determining fetus age if it is conducted before appearing the fetuses’ biological differences (before 22^nd^ week of pregnancy) (6). 

In sonography, fetus age is estimated by BPP, distance of head from femur and length of femur (7). Use of Doppler velocimetry to evaluate fetal-placental and uterine blood provision may be useful in recognizing and assessing IUGR severity. The technique can be studied specially in mother uterine arteries and fetus umbilical and cerebral arteries (8). It may be one of the important predictive factors in fetus hypoxia (9). 

Abel *et al* in their study on value of blood circulation study from different parts of fetus middle cerebral artery, concluded that there is not meaningful difference in studying each of distal, middle and end parts of artery (10). Mu *et al* conducted a study on value of prediction of velocimetry study of middle cerebral arteries in term neonates and concluded that the mentioned study is useful in prediction neonates’ neurological outcomes (11). Analysis of umbilical artery blood gases are used to evaluate hypoxia condition in the born neonates (12). 

The present study aimed at comparing of fetal middle cerebral arteries, umbilical and uterin artery color Doppler ultrasound with blood gas analysis in pregnancy complicated by IUGR.

## Materials and methods

In a descriptive-analytical study, 100 cases of symetric IUGR pregnancies in Tabriz Alzahra Hospital from January 2010 to January 2011 were studied randomly. Results of doppler sonography of fetus middle cerebral arteries, umbilical and uterine artery and umblical ABG were studied in these neonates. IUGR was defined as growth at the 10^th^ or less percentile for weight of all fetuses at that gestational age. This study was approved by Research Ethics committee of Tabriz University of Medical Sciences. 

Written consent was obtained from all the study population. Inclusion criteria were pregnancy between 32-40 weeks, and lack on known abnormalities in fetus. Exclusion criteria were pregnancy with delivery problems including long delivery, pelvic inadaptability and pre rupture of membrane (PROM). One hundred pregnant women were selected based on clinical examinations. According to sonographic studies, they suffered from IUGR and qualified to enter the study. After taking their satisfaction, Doppler sonography and velocimetry date including fetus middle cerebral artery, umbilical and uterine arteries were registered. 

Additionally, delivery conditions such as special events while delivery, type of delivery and neonate’s apgar was registered and fetuses with anomaly were excluded from the study. To analyze umbilical ABG, 1-2^CC^ blood was taken from umbilical artery of umbilical cord in a heparin syringe and immediately sent to the laboratory to be analyzed considering ABG. 

The obtained results were registered. The understudy variables include pregnancy age based on sonography, pregnancy age based on fundal height examination, PCO_2_, pH, PO_2_, type of delivery, neonate apgar, results of Doppler sonography of fetus middle cerebral artery, results of Doppler sonography of umbilical artery, results of Doppler sonography of right and left uterine artery and neonate complications. 

**Statistical analysis**

SPSS^TM^, version 16 is the used statistical software program. Chi-square test was used to evaluate mean comparisons and Mann-Whitney-U test was applied to study the relationship between rank and qualitative variables. The resulted outcomes stated as frequency percentage, mean along with standard deviation and p<0.05 was regarded as the meaningful level.

## Results

In this study, 100 pregnant mothers hospitalized due to IUGR were studied. Mean maternal age was 28±7 years, mean gestational age was 31.79±2.59 weeks and mean growth restriction was 3±2 weeks. Doppler sonography data was studied considering uterine arteries, fetus middle cerebral arteries and umbilical arteries. 

The data was compared with results of ABG analysis. Resistance increasing was observed in right uterine arteries of 37 mothers (resistance index mean=0.74±0.05) and nuch was seen in three cases, while it was normal in 60 mothers. Resistance increasing was observed in left uterine arteries of 36 mothers (resistance index mean=0.76±0.16 and beat index mean=2.16±0.37) and nuch was seen in four cases, while it was normal in 60 mothers. 

Considering umbilical arteries, resistance increasing was observed in 54 patients (resistance index mean=0.88±0.11 and beat index mean=1.60±0.36). It was normal in 46 patients and is accompanied by omission of diastole end stream in 22 patients and reversing of diastole end stream in 3 cases. Resistance decreasing was observed in fetus middle cerebral artery in 51% of cases (resistance index mean=0.66±0.06 and beat index mean=1.15±0.17). In 2% of patients, omission of diastole end stream was seen. Reversing of diastole end stream was not seen in any cases. It was normal in 47% of cases. PCO_2_ and PO_2_ mean were 48.41±9.50 and 26.00±12.34 respectively in the understudy neonates. 

pH mean was 7.28±0.10 in the understudy neonates. It was less than 7 and over than 7.3 in five and 67 cases respectively (Table I). Comparing results of vessels color Doppler with analyses of arterial blood gases demonstrated that disorder in results of arterial blood gases and neonatal acidity (pH<7) were seen in 3 fetuses suffering from disorders of uterine arteries (p=1). 

Disorder in results of arterial blood gases and neonatal acidity (pH<7) were observed in 4 fetuses with disorders of umbilical arteries (p=0.387). Disorder in results of arterial blood gases and neonatal acidity (pH<7) were observed in 1 fetus with disorders of middle cerebral arteries (p=0.192). Relationship between pH and Doppler sonography is observed in table II.

**Table I.** Comparison of ABG characteristics according to Doppler sonography

		**pH**	**Pco**_2_	**Po**_2_	**B/E**
Uterine artery					
	Normal	7.29 ± 0.1	46.89 ± 9.85	26.39 ± 13.69	-6.59 ± 3.82
	Abnormal	7.24 ± 0.11	49.88 ± 9.02	25.63 ± 11.02	-6.87 ± 3.99
Middle cerebral Artery					
	Normal	7.27 ± 0.11	47.68 ± 10.58	25.92 ± 12.64	-6.81 ± 4.69
	Abnormal	7.23 ± 0.1	49.10 ± 8.43	26.08 ± 12.18	-6.66 ± 3.02
Umbilical artery					
	Normal	7.29 ± 0.09	46.69 ± 9.83	26.05 ± 13.14	-6.36 ± 4.20
	Abnormal	7.22 ± 0.12	49.72 ± 9.12	25.96 ± 11.83	-7.01 ± 3.66

ABG: Arterial blood gas

Values are presented as Mean±SD.

**Table II:** Relationship between pH and Doppler sonography

		**pH<7.3 [N (%)]**	**pH>7.3 [N (%)]**	**p-value** ^*^
Uterine artery				0.724
	Normal	32 (32)	17 (17)	
	Abnormal	35 (35)	16 (16)	
Middle cerebral artery				0.358
	Normal	30 (30)	18 (18)	
	Abnormal	37 (37)	15 (15)	
Umbilical artery				0.437
	Normal	27 (27)	16 (16)	
	Abnormal	40 (40)	17 (17)	

*Mann*-*Whitney U test*

## Discussion

The term intrauterine growth restriction (IUGR) has largely replaced the term intrauterine growth retardation. The preferred method for evaluating IUGR is ultra sonographic examination. In their study, Rhee *et al* stated that Doppler sonography in pregnant women play a significant role in evaluating fetus growth condition as well as studying IUGR pregnancies (13). Baschat *et al* in their study on Doppler sonography in IUGR pregnancies suggested that umbilical, uterine and middle cerebral arteries abnormality is seen at higher levels in those fetuses suffering from IUGR (14). 

Mari *et al* stated that evaluating fetus middle cerebral artery provide us useful information required for assessing IUGR fetuses conditions (15). In their study, Sohn *et al* suggested that abnormal results of fetus Doppler sonography are effective factors in appearing abnormalities including IUGR (16). 

Results of the above-mentioned study make it clear that evaluating Doppler sonography of middle cerebral, umbilical and left and right uterine arteries in pregnant women provide us useful information. According to the results of the study, fetus fate and its suffering from IUGR can be predicted. The method can be useful in diagnosing fetal hypoxia because it is a safe, economical and available method. 

In our study, abnormality rate of fetus middle cerebral, umbilical and uterine arteries was observed in IUGR pregnancies. In their study on pH and PO_2_ of neonates suffering from IUGR, Blackwell *et al* stated that PH and PO_2_ level of these neonates is less than that of the normal ones (17). 

Ferrazzi *et al* concluded that PO_2_ and PH level of the neonates with IUGR are lower while PCO_2_ level is higher than neonates with natural growth (18). In our study, means obtained from analysis of umbilical artery blood gases was somehow less than natural rate. But, significant difference was not observed between acidity of these neonates and natural rates. The difference observed between our results with these studies may be due to earlier interventions in our study. 

In our study it was made clear that although difference between this parameter was not meaningful in fetuses with normal and abnormal Doppler sonography of fetus middle cerebral artery, pH and PO_2_ level in fetuses with normal Doppler sonography of fetus middle cerebral artery was higher than other fetuses but PCO_2_ level of fetuses with abnormal Doppler sonography of fetus middle cerebral artery was higher. It approves effects of fetus middle cerebral artery abnormality on parameters of umbilical artery blood gases. 

In a study conducted by Weiner and Robillard in America, it was demonstrated that PO_2_, pH and oxygen saturation in neonates suffering from IUGR was less that control group but their PCO_2_ level was significantly higher than that of the control group (19). Yoshiwura *et al *suggested that evaluating fetus middle cerebral and umbilical arteries using Doppler sonography is useful in early diagnosis of IUGR pregnancies (20). In this study abnormal color Doppler in IUGR fetuses have no significant correlation with umbilical cord blood gas.

## References

[1] Figueras F, Gardosi J (2011). Intrauterine growth restriction: new concepts in antenatal surveillance, diagnosis, and management. Am J Obstet Gynecol.

[2] Orgeig S, Crittenden TA, Marchant C, McMillen IC, Morrison JL (2010). Intrauterine growth restriction delays surfactant protein maturation in the sheep fetus. Am J Physiol Lung Cell Mol Physiol.

[3] Morrison JL, Botting KJ, Dyer JL, Williams SJ, Thornburg KL, McMillen IC (2007). Restriction of placental function alters heart development in the sheep fetus. Am J Physiol Regul IntegrComp Physiol.

[4] Supramaniam VG, Jenkin G, Loose J, Wallace EM, Miller SL (2006). Chronic fetal hypoxia increases activin A concentrations in the late-pregnant sheep. BJOG.

[5] Barth A, Bauer R, Gedrange T, Walter B, Klinger W, Zwiener U (1998). Influence of hypoxia and hypoxia/hypercapnia upon brain and blood peroxidative and glutathione status in normal weight and growth-restricted newborn piglets. Exp Toxicol Pathol.

[6] Ventura W, Nazario C, Ingar J, Huertas E, Limay A, Castillo W (2010). Prenatal sonographic diagnosis of duplicated middle cerebral artery. Fetal Diagn Ther.

[7] Bailão LA, Osborne NG, Rizzi MC, Bonilla-Musoles F, Duarte G, Bailão TC (2005). Ultrasound markers of fetal infection part 1: viral infections. Ultrasound Q.

[8] Piazze J, Gioia S, Cerekja A, Larciprete G, Argento T, Pizzulo S (2007). Doppler velocimetry alterations related to platelet changes in third trimester pregnancies. Platelets.

[9] Abuhamad A (2004). Color and pulsed Doppler in fetal echocardiography. Ultrasound Obstet Gynecol.

[10] Abel DE, Grambow SC, Brancazio LR, Hertzberg BS (2003). Ultrasound assessment of the fetel middle cerebral artery peak systolic velocimetry. Am J Obstet Gynecol.

[11] Mu J, Kanzaki T, Tomimatsu T, Fukuda H, Fujii E, Takeuchi H (2002). Investigation of intraplacental villous arteries by Doppler flow imaging in growth-restricted fetuses. Am J Obstet Gynecol.

[12] Macedonia C, Miller JL, Sonies BC (2002). Power Doppler imaging of the fetal upper aerodigestive tract using a 4-point standardized evaluation: preliminary report. J Ultrasound Med.

[13] Rhee E, Detti L, Mari G (1998). Superior mesenteric artery flow velocity waveforms in small for gestational age fetuses. J Matern Fetal Med.

[14] Baschat AA, Gembruch U, Reiss I, Gortner L, Weiner CP, Harman CR (2000). Relationship between arterial and venous Doppler and perinatal outcome in fetal growth restriction. Ultrasound Obstet Gynecol.

[15] Mari G, Abuhamad AZ, Uerpairojkit B, Martinez E, Copel JA (1995). Blood flow velocity waveforms of the abdominal arteries in appropriate- and small-for-gestational-age fetuses. Ultrasound Obstet Gynecol.

[16] Sohn C, Meyberg G (1995). [Initial experiences with a new color technique: ultrasound angiography]. Zentralbl Gynakol.

[17] Blackwell SC, Moldenhauer J, Redman M, Hassan SS, Wolfe HM, Berry SM (2001). Relationship between the sonographic pattern of intrauterine growth restriction and acid-base status at the time of cordocentensis. Arch Gynecol Obstet.

[18] Ferrazzi E, Bellotti M, Marconi A, Flisi L, Barbera A, Pardi G (1995). Peak velocity of the outflow tract of the aorta: correlations with acid base status and oxygenation of the growth-retarded fetus. Obstet Gynecol.

[19] Weiner CP, Robillard JE (1988). Atrial natriuretic factor, digoxin-like immunoreactive substance, norepinephrine, epinephrine, and plasma renin activity in human fetuses and their alteration by fetal disease. Am J Obstet Gynecol.

[20] Yoshimura S, Masuzaki H, Gotoh H, Ishimaru T (1995). The relationship between blood flow redistribution in umbilical artery and middle cerebral artery and fetal growth in intrauterine growth retardation. Nippon Sanka Fujinka Gakkai Zasshi.

